# Somatic Genome Variations in Health and Disease

**DOI:** 10.2174/138920210793176065

**Published:** 2010-09

**Authors:** I.Y. Iourov, S.G. Vorsanova, Y.B. Yurov

**Affiliations:** 1 National Research Center of Mental Health, Russian Academy of Medical Sciences; 2 Institute of Pediatrics and Children Surgery, Rosmedtechnologii; 3 Moscow City University of Psychology and Education, Moscow, Russia

**Keywords:** Somatic genome variations, aneuploidy, chromosome instability, genomic instability, disease.

## Abstract

It is hard to imagine that all the cells of the human organism (about 10^14^) share identical genome. Moreover, the number of mitoses (about 10^16^) required for the organism’s development and maturation during ontogeny suggests that at least a proportion of them could be abnormal leading, thereby, to large-scale genomic alterations in somatic cells. Experimental data do demonstrate such genomic variations to exist and to be involved in human development and interindividual genetic variability in health and disease. However, since current genomic technologies are mainly based on methods, which analyze genomes from a large pool of cells, intercellular or somatic genome variations are significantly less appreciated in modern bioscience. Here, a review of somatic genome variations occurring at all levels of genome organization (i.e. DNA sequence, subchromosomal and chromosomal) in health and disease is presented. Looking through the available literature, it was possible to show that the somatic cell genome is extremely variable. Additionally, being mainly associated with chromosome or genome instability (most commonly manifesting as aneuploidy), somatic genome variations are involved in pathogenesis of numerous human diseases. The latter mainly concerns diseases of the brain (i.e. autism, schizophrenia, Alzheimer’s disease) and immune system (autoimmune diseases), chromosomal and some monogenic syndromes, cancers, infertility and prenatal mortality. Taking into account data on somatic genome variations and chromosome instability, it becomes possible to show that related processes can underlie non-malignant pathology such as (neuro)degeneration or other local tissue dysfunctions. Together, we suggest that detection and characterization of somatic genome behavior and variations can provide new opportunities for human genome research and genetics.

## INTRODUCTION

The human organism consists of about 10^14^ cells of 210 different types that originate from one zygote and are the result of about 10^16^ mitoses (approximately 45 cellular generations). Moreover, the large number of cellular divisions is required to maintain relatively stable amount of cells in a human body to cover each day’s loss of more than several tens of millions of cells. These numbers make clear that such an “amount of processes” cannot be identically reproduced and, therefore, all the cells of an organism are unlikely to possess identical genomes. 

It appears that the most critical period for somatic mutations to occur is early embryonic development [1, 2]. Characterized by the logarithmic increase in cells (the most dramatic increase of cell numbers in human ontogeny) [2], human embryos are thought to exhibit increased levels of mitotic mutations [3-5]. This is experimentally confirmed by molecular cytogenetic studies of embryonic and fetal cells, which demonstrate high rates of aneuploidy due to mitotic errors correlated with high cell division rate [5, 6]. Therefore, genetically altered cells produced during this ontogenetic period form a basis for organism dysfunction at the following developmental stages [3, 4, 7]. Nonetheless, there is still a possibility that somatic genome variations (SGV) lack adverse effect due to natural selection and clearance of abnormal cells [5, 4, 7, 8].

The genome of a cell is supposed to experience 10^4^-10^5 ^of DNA lesions per day. This is another source (exogenous source) for cellular genome to change and, if remains unrepaired (uncleared), such genomic variations give rise to pathogenic processes (i.e. cancerization) [9]. Although this is a likely process for diseases caused by SGV produced through either genomic instability (GIN) or chromosome instability (CIN) [7-12], it is supposed to be an underlying mechanism of human aging [8, 11].

Despite of numerous attempts to highlight the role of SGV [2-8, 10, 12-22], related phenomena remain largely underappreciated in current biomedical literature. This suggests that an additional attention to SGV is required. Hence, a review of SGV might help to define the contribution to human interindividual diversity in health and disease.

## NATURAL SGV

Since benign genomic variations of somatic genome remain to be poorly described, it is hard to assess the effect of SGV on the non-pathogenic diversity. Thus, no less than 12% of the human genome encompassing disease-associated loci is diversified between two individuals [23]. Although it is difficult to extrapolate these data to cell populations, it can be considered as an indirect evidence for cellular genome to change in a related manner. Fortunately, there are molecular cytogenetic data on SGV manifested at chromosomal level (structural rearrangements, aneuploidy and polyploidy) in early prenatal development. As to other types of genomic variations, including single-base DNA changes, DNA sequence deletion/duplications/inversions, repeat expansions, transposition of mobile DNA elements, copy number variations (CNV), chromosomal miscrodeletions/microduplications (for more details see reviews [15, 24]), their incidence among human fetuses remain largely unknown. SGV detected after birth (non-affected individuals) are mainly referred to low-level mosaic aneuploidy [3, 4, 7]. Mosaic structural genomic rearrangements at chromosomal level are also reported, being, however occasionally detected. Additionally, the best documented SGV are tissue-specific variations of chromosome numbers (aneuploidy) and CNV. Table **1** gathers the data on SGV in normal human tissues (cell types).

The essential problem surrounding the evaluation of SGV is a technological one (for more details see [3, 4, 7, 15, 18, 22, 24]). In other words, some tissues or developmental stages were evaluated using single-cell high-resolution molecular cytogenetic techniques, whereas others were not [22]. Therefore, it is hard to compare different data on SGV. Nevertheless, preimplantation embryos exhibit high rates of SGV manifested at chromosomal (microscopic and submicroscopic) level including aneuploidy, gross structural genomic rearrangements, CNV, segmental duplications. In total, it is estimated that almost 90% of samples have cells with different genomes [6, 25, 26]. The intercellular rate of variations (percentage of abnormal cells) is uninformative because of small amount of cells at this developmental stage [3, 4]. At the next stages of prenatal development, a lesser frequency of SGV is observed, being, still, appreciable and affecting up to 30% of fetuses (aneuploidy) [4, 5, 8]. This is observed in extraembrionic tissues [5, 27] and is suggested to play a key role in normal human placentation [27]. Additionally, no less than 30-35% of cells of the developing human brain and 20% of fetal skin are aneuploid [5, 28]. Finally, fetal ovarian tissues demonstrate a significant increase of mosaic trisomy of chromosome 21 [29]. Further periods of human intrauterine development ascertained through prenatal diagnosis (chorionic villus sampling (CVS) and amniocentesis) show a small rate of SGV [30]. However, it is to note that these periods are rarely evaluated by molecular cytogenetic techniques (singular case-reports only), which are essential for accurate SGV detection [3, 22]. An additional issue of such studies is description of another example of apparently benign tissue-specific SGV in human fetuses referred to as placental mosaicism [31, 32]. Together, SGV appear to be mainly formed during prenatal development and have the potential to give rise to intercellular diversity after birth in health and disease.

Newborns were not thoroughly evaluated in terms of SGV. Furthermore, the only available data on large genomic variations (chromosomal abnormalities and heteromorphism) can be only acquired from papers describing banding studies performed in the end of 70s or beginning of 80s [4, 33, 34]. Chromosomal mosaicism detected by banding cytogenetics in newborns is less frequent than 0.1% [33, 34]. Molecular cytogenetic evaluations of control group in a survey of SGV in autism showed rates of mosaic aneuploidy in blood lymphocytes as 0.73% (autosomes) and 1.11% (chromosome X) [35]. Mosaic structural chromosome rearrangements are extremely rare and are supposed to be detected in a clinical population only [36]. Middle age adults exhibit 1-3% of aneuploid cells in tissues composed of mitotically active cells and less than 1% in the adult human brain, which is mainly composed of post-mitotic cells [10, 28, 37-45]. Natural SGV manifesting as structural rearrangements detectable by banding cytogenetics in blood lymphocytes achieve the rate of 0.6% [40]. Mosaic subtle structural genomic rearrangements and CNV can be tissue-specific in presumably unaffected individuals [46, 47]. Aged human tissues are known to be featured by increased rates of SGV essentially manifesting as low-level mosaic aneuploidy [8, 10, 28, 37-39, 41, 43-45]. In conclusion, three main features of natural human SGV may be highlighted: (i) SGV do contribute to human natural (intercellular) genomic variation; (ii) further studies are strongly required to identify incidence and possible effect of SGV on unaffected human tissues; (iii) SGV have different rates at different ontogenetic stages. The latter suggests a role for SGV in human development and aging.

## SGV AND DEVELOPMENTAL/AGING PROCESSES

Although involvements of SGV in developmental and aging processes are presented in another review published in this Hot Topic Issue (YB Yurov *et al*. Ontogenetic variation of the human genome), we found needful to mention briefly related phenomena. This appears to be important for further delineation of the role of SGV in human diseases and mechanisms of SGV formation. Two kinds of fates of abnormal cells formed during early prenatal development are hypothesized: persistence (increase or stability of rates) and clearance (decrease of rates). The former is supposed to represent a mechanism for SGV-associated diseases (i.e. brain diseases, cancers, mosaic chromosome abnormalities), where as the latter is likely to be a normal process aimed at regulation of cellular population size and to protect against aneuploidization or other unfavorable SGV [4, 5, 7, 8, 47, 48]. Similar processes appear to underlie human aging, including diseases of pathological/accelerated aging [8, 10, 18, 45]. In sum, this suggests that SGV formed during prenatal development are probably responsible for human prenatal mortality and postnatal morbidity. However, SGV originating from somatic mutations after birth are likely to be diseases-causing, as well (as exemplified by studying GIN and CIN in cancers).

## SGV AND HEREDITARY DISEASES

Genomic variations are determined according to DNA sequence size that is involved in a rearrangement [24]. Numerous studies performed during the last decade were focused on genomic variations at DNA sequence level (gene mutations) [2, 3, 13, 19, 24] and copy number variations (CNV) [2, 3, 6, 15, 16, 23, 24, 46]. In this extent, SGV was continuously studied in monogenic syndromes and diseases associated with CNV [2, 19]. Table **2** summarizes current data on SGV contribution to pathogenesis of hereditary diseases caused by gene mutations and CNV.

It is probable that some somatic CNV encompassing these genes are, as yet, undescribed due to extreme rarity of the these conditions (at least some of these conditions) [2, 19]. Additional important issue of somatic gene mutations and CNV is related to explanation of phenotypic difference between cases of the same syndrome due to different expressivity in cases of SGV [19]. Finally, the list of somatic gene mutations and CNV is far from being complete. Furthermore, some of them appear to be benign in a proportion of cases [2].

## SGV AND CHROMOSOME SYNDROMES

The best documented genomic variations are those detected at submicroscopic and microscopic levels (subtle structural genomic rearrangements and chromosomal abnormalities) [3, 15, 24]. As mentioned above, mosaic structural chromosomal rearrangements are rare. There are few population-based cytogenetic studies of these SGV suggesting them to be associated with milder manifestations of the corresponding non-mosaic rearrangement [36]. Mosaic subtle structural chromosome abnormalities (undetectable by banding cytogenetic techniques) are repeatedly reported but the incidence remain to be estimated [47, 49]. Consequently, this review part is primarily focused on numerical chromosome abnormalities (aneuploidy and poliploidy). According to previous review of chromosomal mosaicism [4], mosaic aneuploidy can be divided into three major groups: rare mosaic autosomal aneuploidy (chromosomes 1, 2, 3, 4, 5, 6, 7, 10, 11, 12, 17, and 19); relatively rare mosaic autosomal aneuploidy (chromosomes 14, 15, 16, and 20); frequent mosaic autosomal aneuploidy (chromosomes 8, 9, 13, 18, 21, and 22). Aneuploidy of sex chromosomes is common due to reduced phenotypic effect as to autosomal aneuploidy [3, 4, 15]. The majority of aneuploidy is trisomy or additional sex chromosomes, inasmuch as loss of an autosome leads to intrauterine death at the earliest stages of prenatal development in contrast to loss of chromosomes X and Y [4, 50, 51]. Interestingly, trisomies of chromosomes, rarely involved in aneuploidy in fetuses and liveborn infants (adults), occur at the same rate in preimplantation embryos as mosaic trisomies of other chromosomes [52]. This suggests that mosaic aneuploidy does not possess appreciable effect on the earliest stage of embryonic development. Therefore, the next stages of the development should exhibit high rates of chromosomal mosaicism. This is supported by data on spontaneous abortions, 25% of which are chromosomal mosaics [50]. Additionally, the presence of uniparental disomy in liveborns is considered a confirmation of cleared prenatal mosaicism (confined placental mosaicism) [53]. Chromosomal mosaicism is also associated with asymmetry and skin pigmentary anomalies [54]. For instance, some syndromes featured by congenital asymmetric deformations exhibit unshared distribution of aneuploid or polyploidy cell lines [3, 4, 54].

After birth, mosaic chromosomal abnormalities are essentially identified among individuals with phenotypic manifestation of recognizable aneuploidy (chromosomal) syndromes [3, 4, 15, 55]. However, there are several reports about unaffected individuals with up to 30% of abnormal (aneuploid) cells (reviewed in [3] and [4]). The proportion of mosaics reflects unequal susceptibility of different chromosomes to mitotic non-disjunction [4, 55]. Table **3** shows mosaicism (mitotic non-disjunction) among cases of aneuploidy in humans.

Another well-described examples of SGV associated with chromosomal abnormalities (chromosomal syndromes), are small supernumerary marker chromosomes. Over 50% of cases demonstrating these chromosomal rearrangements can exhibit mosaicism, including tissue-specific forms [4, 56-58]. More precise information about SGV and marker chromosomes is presented in another review of this Hot Topic Issue (T Liehr *et al*. Somatic mosaicism in cases with small supernumerary marker chromosomes).

SGV demonstrate a diminished clinical effect of chromosome abnormalities [3, 4, 7, 15]. However, cancers, which are all caused by somatic mutations, are primarily associated with cellular (tissular) pathology [11, 13, 14, 17, 20, 41, 42]. Taking into account these facts, a hypothesis suggesting that SGV manifested as somatic chromosomal mutations (the commonest type of SGV) can be a source or a susceptibility factor for complex human diseases was proposed [3, 4, 7, 8, 12, 15, 48].

## SGV AND COMPLEX DISEASES

Currently, SGV have been described in individuals with brain diseases (psychiatric and neurodegenerative) [2-4, 7, 15-17, 19, 22, 35, 55, 59-80], autoimmune diseases [81-83], congenital heart diseases [84] and cancer [9, 11, 13-15, 20, 85]. All these data is summarized by Table **4**.

Theoretically, any mutation can be somatic leading, thereby, to a disease [3]. However, since genomic variations are classically thought to be a result of germline mutations or genomic rearrangements [13, 86], SGV contributions to human morbidity is poorly appreciated. In contrast, complex diseases appear to be likely associated with somatic mutations as to monogenic syndromes and genomic disorders, being commonly associated with tissue-specific (or even “subtissue-specific”) pathology without any additional dysfunctions in other unaffected tissues [3, 4, 7, 12]. Moreover, SGV are able to explain some important features of complex disease-causing genetic alterations such as environmental effects or specific male-to-female ratios [4, 8, 82, 87-89]. Finally, to get an integral view of how SGV and other disease-causing processes interplay with each other, formation mechanisms of somatic mosaicism are to be established.

## SGV FORMATION MECHANISMS

Although formation of somatic mutations (aneuploidy and polyploidy) was the major focus of numerous studies in fields of cell biology, genetics and oncology, it is still incompletely understood. A number of neonatal mosaics and cases of tissue-specific mosaicism is suggested to result from trisomy rescue due to placental mosaicism [3, 4, 27, 31, 53], but it appears to be not the case of spontaneous abortions [50]. In neurodegenerative and aging diseases, somatic aneuploidy is probably the result of cellular natural selection — abnormal cells possess the potential to survive and to proliferate [8, 10, 45]. Studies of somatic cell division (mitosis) suggest that mitotic non-disjunction and anaphase lagging are two main mechanisms for post-zygotic aneuploidy formation (aneuploidization). Numerous intracellular processes are assumed to be involved in improper somatic cell divisions producing GIN and CIN. Among these are defects in kinetochore apparatus, centrosomes amplification, genetic and epigenetic alterations to mitotic checkpoint genes (aneuploidy/polyploidy) as well as abnormal DNA reparation and replication (structural alterations to chromosomes, aneuploidy, polyploidy) [3, 11, 45, 90-96]. Polyplodization followed by multipolar cellular divisions are also hypothesized to be a major contributor to somatic aneuploidization associated with human diseases [96]. Nonetheless, there is still a lack of an integrated view on SGV formation.

## CONCLUDING REMARKS

In a previous issue of *Current Genomics*, we have hypothesized that uniqueness of a cell is achieved *via *SGV [3]. Single-cell gene expression studies showing that there is no an average cell, because each one has own unique epigenetic profiling (or epigenome) [21, 97]. Here, we would like repeatedly adopt this idea to the cellular genome. Four years after the first postulation [3], important additional data on SGV contribution to normal and pathological human biodiversity have been accumulated. It was found that early human prenatal development was defined as a major source for SGV [5, 6]. It has been shown that neurodegeneration is mediated by GIN and CIN like in cancer in such devastative genetic brain diseases as ataxia-telangiectasia and Alzheimer’s disease [10, 45]. Several psychiatric diseases (autism and schizophrenia) have been associated with mosaic (somatic) aneuploidy [35, 44]. Additionally, very recent reports provided by others groups of researchers showed SGV implicated in the normal and abnormal brain physiology and aging [98-100]. These results provide essential evidences that neuronal DNA variation is a new feature of the human brain, which may contribute to neural diversity in normal and pathophysiological states and differences amongst individuals. Together, one can conclude that SGV research has proven itself sufficiently to become an important biomedical field that would help to understand cellular and molecular processes determining human life- and health-span.

## Figures and Tables

**Table 1:** SGV in Normal Human Tissues

Tissue/Cell type	Type of SGV	Description	Key Refs
Preimplantation embryos	Aneuploidy	15-91% of samples (mean is about 50%)	[6, 25, 26]
	Structural rearrangements, aneuploidy, CNV*, segmental duplications, uniparental disomy	>90 %of samples (83 % — aneuploidy)	[6]
**Embryos/Fetuses (7-12 weeks)**			
Cytotrophoblasts	Aneuploidy	20-60% of cells	[27]
Brain	Aneuploidy	1.45% of cells ^	[5, 28]
Chorionic villi	Aneuploidy	0.98% of cells ^	[5]
Skin	Aneuploidy	0.82% of cells^	[5]
Ovarian tissue	Trisomy of chromosome 21 (aneuploidy)	Statistically significant increase of aneuploid cells	[29]
Prenatal diagnosis: CVS** or Amniocentesis			
Amniocytes	Aneuploidy	0.25% of samples	[30]
Chorionic villi/Placenta	Aneuploidy	1-2% of samples	[31, 32]
**Newborns/Children**			
Blood lymphocytes	Aneuploidy	>0.1% (clinical population?)	[4, 33, 34]
Blood lymphocytes	Aneuploidy	0.73% (autosomes) and 1.11% (chromosome X) of cells — unaffected population^	[35]
Blood lymphocytes	Structural rearrangements	0.01% (clinical population?)	[36]
**Adults (middle age)**			
Blood lymphocytes	Aneuploidy	1-3% of cells^	[37-39]
Blood lymphocytes	Structural rearrangements	0.6% of cells	[40]
Skin fibroblasts	Aneuploidy	2.2% of cells^	[41]
Liver	Aneuploidy	3% of cells^	[42]
Brain	Aneuploidy	0.3-0.9% of cells^	[10, 28, 43-45]
Brain	CNV*	Tissue-specific CNV; amount of cells and percentage of samples was not available	[46]
Skin			
Heart			
Kidney			
Liver			
T-lymphocytes	Subtle structural rearrangements or CNV	Tissue-specific mosaicism probably originating from developmental chromosome instability	[47]
Imortalized B lymphoblastoid cells			
Skin fibroblasts			
**Adults (aged individuals)**			
Blood lymphocytes	Aneuploidy	1-2% (autosomes) and 4-7% (chromosome X) of cells^	[37, 39]
Skin fibroblasts	Aneuploidy	4.4% of cells^	[41]
Brain	Aneuploidy	0.3-0.9% of cells^	[10, 28, 43-45]

* — copy number variations;

** — chorionic villus sampling;

^ — per chromosome.

**Table 2:** SGV and Hereditary Diseases Demonstrating Somatic Gene Mutations or CNV (in Parts Adopted from [2] and [16])

Locus	Disease	Gene	CNV	Gene Mutations
1q21.2	Progeria	*LMNA*	-	+
1q44	Chronic infantile neurologic cutaneous articular	*CIAS1*	-	+
2p22p21	Hereditary spastic paraplegia	*SPG4*	-	+
2q24	Myoclonic epilepsy	*SCN1A*	-	+
2q31	Ehlers Danlos Syndrome IV	*COL3A1*	-	+
3p25	von-Hippel-Lindau Disease	*VHL*	+	+
3q13.3q21	Hypocalcemia	*CASR*	-	+
3q27	EEC	*p63*	-	+
4p16.3	Skeletal disorders (syndromes)	*FGFR3*	-	+
4p12	Congenital central hypoventilation	*PHOX2B*	-	+
4q35	Facioscapulohumeral muscular dystrophy	D4Z4*	+	?
5q13	Infantile spinal muscular atrophy	*SMN1*	-	+
6p21	Cleidocranial dysplasia	*RUNX2*	-	+
7q22.1	Osteogenesis imperfecta	***COL1A2***	-	+
8q12.1	CHARGE syndrome	*CHD7*	?	+
9q22	Loeys-Dietz	*TGFBR2*	-	+
11p15.5	Costello syndrome	*HRAS*	-	+
11p15.1	Neonatal diabetes	*KCNJ11*	-	+
12q13	Epidermolysis bullosa simplex	*KRT5*	-	+
12q24.1	Phenylketonuria	*PAH*	-	+
13q14	Retinoblastoma	*RB*	+	+
14q24.3	Alzheimer disease, early-onset	*PS1*	-	+
15q21.1	Marfan	*FBN1*	-	+
16p13	Tuberous Sclerosis	*TSC2*	+	+
16p13	Rubinstein-Taybi Syndrome	*CREBBP*	+	?
17q11	Neurofibromatosis 1	*NF1*	+	+
17q21.31	Osteogenesis imperfecta	*COL1A1*	-	+
17q24	Campomelic dysplasia	*SOX9*	+	+
22q11.2	Several hereditary syndromes	*MYH9*	+	+
Xp22.2p22.1	X-linked hypophosphatemia	*PHEX*	-	+
Xp22.13	X-linked mental retardation (syndromic/nonsyndromic)	*ARX*	-	+
Xp21	Duchenne muscular dystrophy	*DMD*	+	+
Xp21	Chronic granulomatous disease	*CYBB*	+	+
Xp21.1	Ornithine transcarbamylase deficiency	*OTC*	-	+
Xp21.1	Retinitis pigmentosa	*RPGR*	-	+
Xp11.3	Retinitis pigmentosa	*RP2*	-	+
Xq11q12	Androgen insensitivity	*AR*	-	+
Xq26q27.2	Lesch-Nyhan	*HPRT1*	-	+
Xq27	Hemophilia B	*F9*	-	+
Xq28	Hemophilia A	*F8*	+	+
Xq28	Incontinentia pigmenti	*IKBKG*	+	+
Xq28	Mucopolysaccharidosis II	*IDS*	-	+
Xq28	Otopalatodigital syndrome	*FLNA*	-	+
Xq28	Rett syndrome (males and females) and a set of other neurodevelopmental diseases (syndromic/nonsyndromic)	*MECP2*	+	+
Xq28	X-linked dyskeratosis congenita	*DKC1*	+	+
Xq28	X-linked mental retardation	*SLC6A8*	-	+

* — non-coding DNA sequences (repeats).

**Table 3:** Mosaic Cases Among Common Aneuploidies (in Parts from [3, 4, 15, 55])

Aneuploidy	Cases of Mosaicism/Mitotic Non-Disjunction	Incidence	Disease
Trisomy of chromosome 2	7%	unknown	—
Trisomy of chromosome 7	57%*	unknown	—
Trisomy of chromosome 8	50% *	>100 cases reported	Trisomy 8
Trisomy of chromosome 13	1%	1:6000-1:29000	Patau syndrome
Trisomy of chromosome 14	8%	~25 cases reported	Trisomy 14
Trisomy of chromosome 15	None	~10 cases reported	—
Trisomy of chromosome 16	None	~10 cases reported	—
Trisomy of chromosome 18	8%	1:7000	Edwards syndrome
Trisomy of chromosome 21	5%	1:600	Down syndrome
Trisomy of chromosome 22	2%*		Cat eye syndrome (?)
Monosomy of chromosome X	38%*	1:2000 (females)	Turner sydnrome
Trisomy of chromosome X	20%	1:1000 (females)	Trisomy X
47,XXY	9%	1:500 (males)	Klinefelter syndrome
47,XYY	16%	1:800 (males)	Double Y syndrome

* — postnatal cases suggested to be all mosaic.

**Table 4:** SGV in Complex Human Diseases

Diseasee	Type of SGV	Key Refs
**Brain diseases (psychiatric)**		
Learning disability/Mental retardation	Gene mutations, CNV mosaic aneuploidy	[ 2-4, 7, 15-17, 19, 22, 55]
Autism	Mosaic structural/numerical chromosomal abnormalities: Partial tetrasomy 3q Ring chromosome 14 Rearrangements of 15pter-q13.2 Ring chromosome 17 Structural abnormalities + ring chromosome 18 Mosaic deletion 20p	[59-61] [62] [63] [64, 65] [66] [67, 68] [69]
	Mosaic aneuploidy (~16% of cases)	[35]
	Fragile sites	[70, 71]
Schizophrenia	Mosaic sex chromosome aneuploidy (blood lymphocytes)	[72-76]
	Low-level mosaic aneuploidy of chromosomes 1, 18 and X in the diseased brain	[12, 44]
	Fragile sites	[7, 77]
**Brain diseases (neurodegenerative)**		
Alzheimer’s disease	Gene mutations	[78]
	Mosaic aneuploidy of chromosome 21 in the diseased brain	[10]
Huntington’s disease	Gene mutations (trinucleotide repeat expansion) including brain-specific mutations	[79]
Friedreich ataxia	Gene mutations (trinucleotide repeat expansion)	[80]
Ataxia-telangiectasia	Mosaic aneuploidy and chromosome 14-specific breaks/additional rearranged chromosomes	[45]
**Autoimmune diseases**		
Primary immune deficiencies	Revertant somatic mosaicism	[81]
Primary biliary cirrhosis	Mosaic monosomy of chromosome X	[82]
Systemic sclerosis	Mosaic monosomy of chromosome X	[83]
Autoimmune thyroid disease		
**Heart disease**		
Congenital heart diseases	Gene mutations	[84]
	Chromosomal abnormalities/syndromes (?)	[3, 17, 55,]
**Cancers**		
Almost all types of cancers	Almost all cancers are caused by different types of SGV including aneuploidy/polyploidy; balanced and unbalanced structural chromosomal/genomic (subtle and gross) rearrangements; gene amplifications; telomere shortening; microsatellite instability; gene mutations;	[9, 11, 13-15, 20, 85,]
